# Prevalence and Association of Obesity with Self-Reported Comorbidity: A Cross-Sectional Study of 1321 Adult Participants in Lasbela, Balochistan

**DOI:** 10.1155/2017/1076923

**Published:** 2017-10-12

**Authors:** I. Khan, Z. Ul-Haq, A. S. Taj, A. Z. Iqbal, S. Basharat, B. H. Shah

**Affiliations:** ^1^Institute of Public Health & Social Sciences, Khyber Medical University, Peshawar, Pakistan; ^2^Institute of Public Health, Quetta, Balochistan, Pakistan; ^3^Institute of Health and Wellbeing, University of Glasgow, Glasgow G12 8RZ, UK; ^4^Peshawar Institute of Medical Sciences, Peshawar, Pakistan; ^5^Health Services Academy, Islamabad, Pakistan

## Abstract

Association of fatness with chronic metabolic diseases is a well-established fact, and a high prevalence of risk factors for these disorders has increasingly been reported in the third world. In order to incorporate any preventive strategies for such risk factors into clinical practice, decision-makers require objective evidence about the associated burden of disease. A cross-sectional study of 1321 adults from one of the districts of Balochistan, among the most economically challenged areas of Pakistan, was carried out for the measures of fatness and self-reported comorbidities. Body mass index (BMI), waist circumference (WC), and waist-to-hip ratio (WHR) were measured and demographic information and self-reported comorbidities were documented. The prevalence of obesity was 4.8% (95% CI: [3.8, 6.1]) and 21.7% (95% CI: [19.5, 24.0]), as defined by the World Health Organization (WHO) international and Asia/Asia-Pacific BMI cut-offs, respectively. The proportion exhibiting comorbidity increased with increasing levels of fatness in a dose-response relationship (*p* value < .001). An interaction of weight status with gender was observed to produce a significantly (*p* = .033) higher comorbidity among overweight women (odds ratio (OR) = 6.1 [1.2, 31.7]) compared with overweight men (OR = 1.1 [0.48, 2.75], *p* = .762).

## 1. Introduction

Globally, the prevalence of obesity more than doubled between 1980 and 2014. Around 1.9 billion (39%) adults, 38% of men and 40% of women, have been reported to have weights above the normal range. Of these, over 600 million (13%) were found to be obese (11% of men and 15% of women) [1]. This obesity epidemic, previously thought to be a burden of affluent societies, has been noticed to have reached the low- and middle-income countries as a result of the epidemiologic transition [2–4].

Developing countries have been noted to be facing a dual burden of undernutrition and overnutrition simultaneously, exerting substantial strains on the already overburdened health systems [4]. The prevalence of overweight and obesity has increased severalfold in Asia, especially in South Asia [5], over the past few decades, with the extent varying between countries [6], although not very different from that in the United States [7]. Pakistan stands eighth among the 10 countries hosting half of the 693 million obese individuals in the world: USA, China, India, Russia, Brazil, Mexico, Egypt, Pakistan, Indonesia, and Germany [8].

Obesity is a major risk factor for a range of chronic disorders such as cardiovascular diseases (CVD), hypertension, type 2 diabetes mellitus (DM), hypercholesterolemia, osteoarthritis (OA), major depression [9], and certain cancers (CA) [8]. Globally, 23% of CVD, 44% of type 2 DM, and 7–41% of certain cancers are attributable to overweight and obesity, with a major share from developing countries [5].

Obesity has been found to be associated with at least as much morbidity as poverty, smoking, and alcoholism, despite receiving lesser attention in clinical practice and public health domains [10]. In Asia, there is a general paucity of research on obesity using metrics other than BMI, and even for BMI, the cut-off values used are not Asia-Pacific-specific as recommended by the WHO.

The plight of multiple comorbid states has been reported to afflict up to 50% of adult populations [11], with the estimates varying from 13% to 95%  [12]. A palpable lack of research focus regarding the overall burden of this important health problem as well as the balance of inquiry regarding geographic and diagnostic entities has been highlighted, especially for the relatively less disadvantaged tiers of populations in the Eastern Mediterranean region [13].

The aim of this study was to determine the prevalence of obesity and its association with self-reported comorbidity by using different anthropometric measures: BMI (both WHO international and Asian cut-offs), waist circumference (WC), and waist-to-hip ratio (WHR). We also investigated whether the associations varied by gender among a representative sample of adults from the population of the Lasbela District, Balochistan. 

## 2. Methods

### 2.1. Sample Size

Assuming a 15% prevalence of obesity in Pakistan [14], a sample size of 1225 individuals would estimate the true population proportion of obese persons with a 2% margin of error at 95% confidence level. Adding a 10% nonresponse rate, a sample size of 1347 was targeted.

### 2.2. Data Source

Data were collected using multistage stratified random sampling [15]. Out of a sampling frame of 30,000 households from 22 union councils treated as strata, a simple random sample of 270 households and 1321 individuals was selected after exclusion of nonresponders, with a number proportionate to the population from each union council. A list of households in each union council was obtained from the local census offices.

### 2.3. Data Collection

Data for all persons 18 years of age or older, excluding pregnant females, were collected. Face-to-face interviews were conducted by staff specially trained in the measurement process and filling the data collection instrument. The information gathered consisted of demographics (age, gender, education, monthly income, occupation, marital status, and smoking status) and self-reported physician-diagnosed comorbidity (type 2 DM, CVD, hypertension, hypercholesterolemia, OA, and CA). Anthropometric measurements including height, weight, WC, and hip circumference (HC) were taken, with standard operating procedures. Each measurement was taken three times in tandem and then the mean was calculated. BMI was calculated by dividing weight in kilograms (measured after removal of shoes and heavy outer clothing using a CAMRY weighing scale) by the square of height in meters (measured without shoes using a nonstretchable tape). WHR was calculated by dividing WC (measured by a nonstretchable tape at the level of the umbilicus) by hip circumference (measured at the widest point using the same tape) [9].

### 2.4. Operational Definitions

#### 2.4.1. Comorbidity

Within the context of this study, comorbidity is defined as a self-report of a doctor's diagnosis or taking medication prescribed by a doctor for one or more of the following conditions: CVD (coronary heart disease or stroke), hypertension, hypercholesterolemia, type 2 DM, osteoarthritis, or cancer [9].

#### 2.4.2. Obesity

 
*Body mass index* (BMI) based international cut-offs: underweight (<18.5 kg/m^2^), normal weight (18.5–24.9 kg/m^2^), overweight (25.0–29.9 kg/m^2^), and obese (≥30 kg/m^2^).
  The World Health Organization (WHO) recommended cut-offs for Asia and Asia-Pacific region: underweight (<18.5 kg/m^2^), normal weight (18.5–22.9 kg/m^2^), overweight (23.0–24.9 kg/m^2^), and obese (≥25 kg/m^2^) [16].
 
*Waist circumference* (WC) based cut-offs: normal weight (<94 cm), overweight (94–101.99 cm), and obese (≥102 cm) for men; normal weight (<80 cm), overweight (80–87.99 cm), and obese (≥88 cm) for women.
 
*Waist-to-hip ratio* (WHR) based cut-offs: normal weight (<0.90), overweight (0.90–0.99), and obese (≥1) for men; normal weight (<0.80), overweight (0.80–0.84), and obese (≥0.85) for women [17].

### 2.5. Ethical Considerations

This study was conducted after approval from the Ethical Board of the Khyber Medical University, Peshawar (KMU-EB). Consent was taken from the participants on a consent form written in English and Urdu and after explaining to them the elements of the informed consent, their autonomy, confidentiality, right to withdraw at any time they felt uncomfortable, measurement process, and the whole purpose of the study.

## 3. Statistical Analysis

Chi-square (*χ*^2^) tests are used for cross-tabulation analyses. Associations among dichotomized anthropometric measures and comorbidity are assessed by means of univariate and multivariate logistic regression models. Odds ratios (OR) with 95% confidence intervals (CI) are reported and receiver operating characteristic curves presented for these analyses. In the multivariate model, possible confounding factors, that is, gender, age, marital status, education, monthly income, occupation/work status, and smoking status, are adjusted for. Significance of interactions in logistic regression is assessed using Wald's test. Stata version 12.1 (StataCorp, College Station, Texas) has been used to carry out analyses. All tests of statistical significance are two-tailed with an alpha of .05.

Cohen's *d* and *r*-squared are reported as measures of effect size for *t*-tests, Cramér's *V* for chi-square tests, *r* for two-sample tests of proportions, and OR with CI and area under the receiver operating characteristic curve for logistic regression analyses.

## 4. Results

Complete data were available for 1321 persons. With 26 nonresponders, the response rate was 98%. Compared with the participating family members, the nonresponders did not differ in any significant way.

Of the 1321 participants, 659 (49.89%) were males. Mean (M) age of the participants was 32.21 years, with a standard deviation (SD) of 12.83 years.

Mean BMI, WC, and WHR were 22.22 kg/m^2^ (SD: 4.14 kg/m^2^), 79.61 cm (SD: 10.86 cm), and 0.87 (SD: 0.04), respectively.

**Figure 1:**
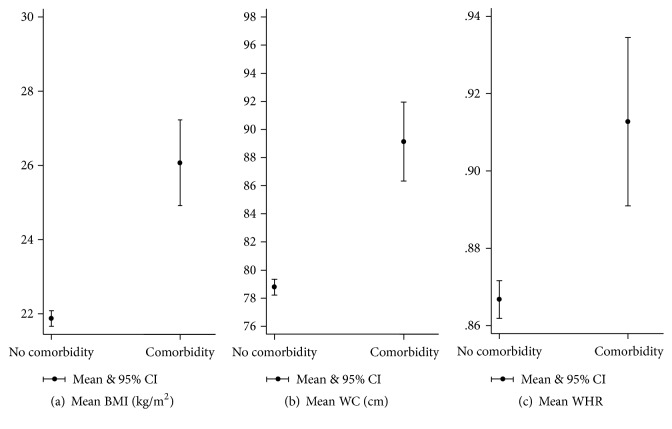
Means of anthropometric measurements* (overall)* with having self-reported comorbidity.

Mean BMI, WC, and WHR were all significantly different between those who had one or more comorbid conditions and those without any comorbidity (*t*(1319) = −10.3915, −9.7439, and −5.0954; all *p* values < .001; *d* = 0.72, 0.99, and 0.52; *r*^2^ = 0.08, 0.07, and 0.02, resp.) (Figure 1).

**Figure 2:**
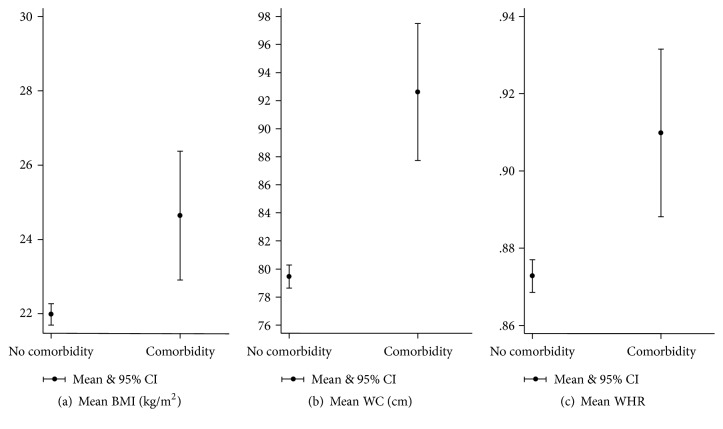
Means of anthropometric measurements* (men)* with having self-reported comorbidity.

Among men, mean BMI, WC, and WHR were all significantly different between those who had one or more comorbid conditions and those without any comorbidity (*t*(1319) = −4.5358, −7.9175, and −4.3972; all *p* values < .001; *d* = 0.69, 1.2, and 0.67; *r*^2^ = 0.03, 0.09, and 0.03, resp.) (Figure 2).

**Figure 3:**
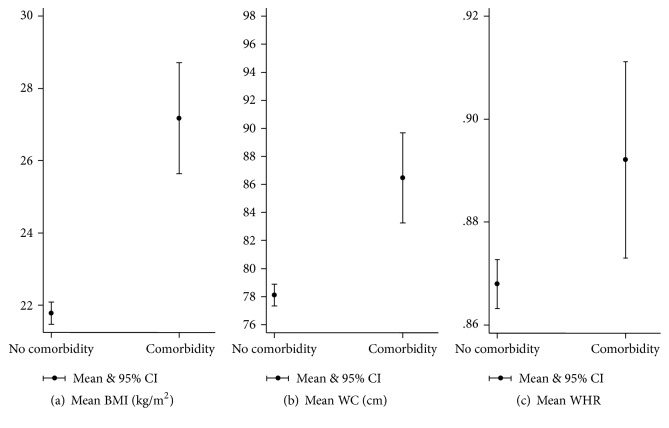
Means of anthropometric measurements* (women)* with having self-reported comorbidity.

Among women, mean BMI, WC, and WHR were all significantly different between those who had one or more comorbid conditions and those without any comorbidity (*t*(1319) = −9.7182, −6.1836, and −2.9383; *p* values < .001, <.001, and .003; *d* = 1.3, 0.083, and 0.4; *r*^2^ = 0.12, 0.06, and 0.01, resp.) (Figure 3).

While WC differed significantly between men (M = 80.4 cm, SD = 11.4 cm) and women (M = 78.8 cm, SD = 10.3 cm) (*t*(1319) = 2.5360, *p* = .011, *d* = .14, *r*^2^ = .005), BMI and WHR did not show such difference (*p* values: .667 and .901, resp.).

The prevalence of obesity was 4.84% (95% CI: [3.8, 6.1]) by international cut-offs and 21.73%  [19.5,24.0] on Asian cut-offs of BMI. On WC measurement and WHR measurement, 10.9%  [9.3,12.7] and 44.4%  [41.7,47.2] were obese, respectively. An obese individual was more likely to be ≥45 years of age (two-sample test of proportions: *z* = −6.2641, *p* < .001, *r* = −0.08), married (*z* = −3.5053, *p* < .001, *r* = −0.1), uneducated (*z* = 2.6437, *p* = .008, *r* = 0.07), and a smoker (*z* = −2.9285, *p* = .003, *r* = −0.08). A person with one or more comorbidities, compared to those without one, was more likely to be female (*z* = −4.7976, *p* < .001, *r* = −.13), forty-five years of age or older (*z* = −15.1801, *p* < .001, *r* = −.42), obese (*z* = −8.3415, *p* < .001, *r* = −.23), uneducated (*z* = 6.1805, *p* < .001, *r* = −.17), and current or ex-smoker (*z* = −3.0239, *p* = .0025, *r* = −.08).

Overall, 8.02%  [6.6–9.6%] of the participants reported themselves as having one or more comorbidities, among whom 43.4%  [33.8,53.4] were men and 56.6%  [46.6,66.2] were women. The difference in proportion of the two genders among those who reported comorbidity was statistically not significant (two-sample test of proportions: *z* = −1.9219, *p* = .06, *r* = −0.13).

The prevalence of various comorbid states, with 95% confidence intervals, was as follows: cardiovascular disease, 1.7%  [1.0,2.5]; hypertension, 5.3%  [4.2,6.6]; type 2 diabetes mellitus, 2.2%  [1.5,3.1]; hypercholesterolemia, 0.9%  [0.5,1.6]; and osteoarthritis, 4.2%  [3.2, 5.4]. None of the participants reported any malignancy.

All of the following variables were significantly associated with self-reported comorbidity: obesity, using both international and Asian cut-offs for BMI (*χ*^2^ (3, *N*: 1321) = 99.16 and 72.26, both *p* values < .001, *V* = 0.27 and .23, resp.), obesity as defined by WC (*χ*^2^ (2, *N*: 1321) = 113.70, *p* < .001, *V* = 0.29), obesity as defined by WHR (*χ*^2^ (2, *N*: 1321) = 21.32, *p* < .001, *V* = 0.13), age (*χ*^2^ (3, *N*: 1321) = 255.18, *p* < .001, *V* = 0.44), marital status (*χ*^2^ (1, *N*: 1321) = 21.48, *p* < .001, *V* = 0.13), educational status (*χ*^2^  (1, *N*: 1321) = 38.22, *p* < .001, *V* = −0.17), and smoking status (*χ*^2^ (2, *N*: 1321) = 9.42, *p* = .009, *V* = 0.08) (Table 1).

In overall multiple logistic regression analysis, after adjusting for potential confounders, that is, gender, age, marital status, education, monthly income, occupation/work status, and smoking status, the association between obesity (as defined by international and Asia-specific cut-off values of BMI) and comorbidity was significant (odds ratios (OR) = 5.75 [2.84,11.64] and 2.87 [1.66,4.97], resp., both *p* values < .001). Overweight was significantly associated with comorbidity only as defined by international cut-offs only (OR = 1.89 [1.10,3.26], *p* = 0.021).

Association between obesity as defined by waist circumference and comorbidity was significant (OR = 3.76 [2.06,6.85], *p* < .001). However, obesity as defined by waist-to-hip ratio did not show a significant association with comorbidity (OR = 1.72 [.63,4.72], *p* = .292) (Table 2).

**Figure 4:**
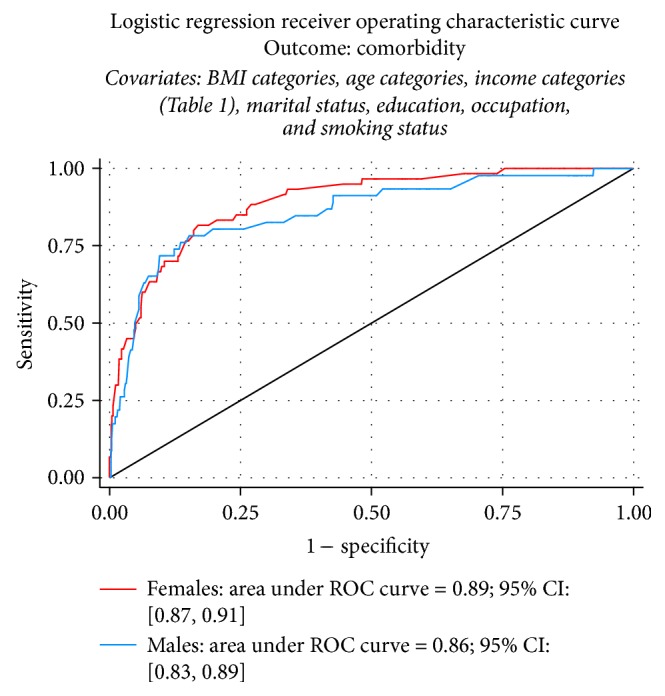
Areas under the ROC curve from logistic regression for males and females.

In gender-specific multiple logistic regression analysis, the association between obesity and comorbidity for men was significant for international BMI cut-offs (OR = 8.49 [2.50,28.81], *p* = .001) but not for Asian cut-offs (OR = 1.96 [.86,4.48], *p* = .109); for women, the association was significant for both cut-offs (OR = 5.19 [2.03,13.26], *p* = .001; OR = 3.73 [1.68,8.29], *p* = .001, resp.) (Figure 4).

The international cut-offs defined overweight was significantly associated with the outcome of comorbidity for women only (OR = 2.64 [1.23, 5.67], *p* = .013), an association not observed in men or with the Asian cut-offs of BMI for women.

Obesity as defined by waist circumference was significantly associated with comorbidity for men as well as women (OR = 6.38 [2.24,18.19], *p* = 0.001; OR = 3.38 [1.49,7.64], *p* = .004, resp.). As defined by waist-to-hip ratio, obesity did not show significant association for either sex.

The overweight category by both cut-offs for BMI showed an interaction with gender to produce significant association (OR: 6.05 [1.16,31.74], *p* = .033) with the outcome of comorbidity for women but not men (OR = 1.14456 [0.48,2.75], *p* = .762).

There was a significant linear trend of increasing comorbidities with progressively increasing weight categories, both by international and by Asia/Asia-Pacific cut-offs (Cuzick's tests for linear trend:* z* = 8.83 and 7.73, resp.,* p* values < .001).

## 5. Discussion

Worldwide, obesity has been reported to incur a twofold increase in the risk of diabetes, hypertension, heart failure, ischemic stroke, ischemic heart disease, and osteoarthritis [18–22]. Our study has found a significant dose-response relationship between increasing comorbidities and increasing weight.

The association has important implications for public health planning and management as health effects of obesity at individual and community levels manifest themselves through these comorbid states, which we have shown to be increasing in direct proportion with increasing weight.

Thirty-seven percent [34.4,39.7] of the participants in this study were either overweight (15.3%  [13.4,17.3]) or obese (21.7%  [19.5,24.0]), according to the recommended BMI cut-offs for Asian populations. Twenty percent [15.40,24.95] of the obese individuals had one or more comorbid conditions.

Our results show a significantly higher prevalence of obesity among those who were 45 years of age or older, married, uneducated, and smokers. Such associations have been reported by various studies across the world including Asia and South Asia [23].

We have found the prevalence of obesity to be 21.7%  [19.5,24.0] and that of underweight to be 17.8%  [15.7,19.9], highlighting the simultaneous burden of the two extremes [4]. The prevalence of underweight that we found is higher than that reported in Pakistan previously (12.3%) [14], probably a reflection of the locale specific economic realities.

Our finding of a significant association of obesity with comorbidities has been reported previously [24] but the levels of different anthropometric measurements at which the participants reported comorbidity in our study were lesser compared to those reported in USA and Europe, probably because Asians are known to carry greater body fat content for a given BMI [25].

This study has shown that the association between BMI (international cut-offs) and self-reported comorbidity differs by the gender; while only obese, not overweight, men were significantly more likely to report comorbidity, both overweight and obese women showed such association. Overweight men, compared with overweight women, have been found to report relatively better quality of life, better psychological health, and more happiness [26–28].

### 5.1. Strengths and Limitations of the Study

The sampling technique for this study was robust enough to ensure representativeness of the sample and external validity. Different anthropometric measures of obesity as well as various cut-off values were used to provide a broader range of assessment for measures of fatness. Anthropometric measurements were done by trained staff rather than relying on self-reported obesity.

Other than being subject to various biases known to beset cross-sectional studies, causality cannot be inferred from the reported associations from this study.

## 6. Conclusion

Comorbidities increase with increasing weight in a dose-response relationship. With the changing social structure and lifestyles in the area, the problem can only get worse with time if not addressed by healthcare planners.

## 7. Recommendations

In order to reduce the healthcare costs, effective preventive strategies have to be put in place at various tiers of healthcare delivery systems.

Additional research with longitudinal design is needed to define the temporal characteristics of association between obesity and its associated comorbidities.

## Figures and Tables

**Table 1:** Characteristics of the participants by self-reported comorbidity.

	Self-reported comorbidity			*p* value
	No		Yes	
	*N* = 1215 (91.98%)		*N* = 106 (8.02%)	
	*N* (%)		*N* (%)	
*Gender*				
Men	613 (93.02)		46 (6.98)	0.163
Women	602 (90.94)		60 (9.06)	
*Age (years)*				
18–30	758 (98.06)		15 (1.94)	<0.001
31–44	299 (95.22)		15 (4.78)	
45–59	112 (73.68)		40 (26.32)	
≥60	46 (56.10)		36 (43.90)	
*BMI category (international cut-offs)*				
Underweight	226 (96.58)		8 (3.42)	<0.001
Normal weight	759 (94.88)		41 (5.12)	
Overweight	189 (84.75)		34 (15.25)	
Obesity	41 (64.06)		23 (35.94)	
*BMI category (Asian cut-offs)*				
Underweight	226 (96.58)		8 (3.42)	<0.001
Normal weight	572 (95.65)		26 (4.35)	
Overweight	187 (92.57)		15 (7.43)	
Obesity	230 (80.14)		57 (19.86)	
*WC category*				
Normal weight	903 (95.35)		44 (4.65)	<0.001
Overweight	212 (92.17)		18 (7.83)	
Obesity	100 (69.44)		44 (30.56)	
*WHR category*				
Normal weight	587 (95.14)		30 (4.86)	<0.001
Overweight	98 (83.76)		19 (16.24)	
Obesity	530 (90.29)		57 (9.71)	
*Marital status*				
Unmarried	483 (96.41)		18 (3.59)	<0.001
Married	732 (89.27)		88 (10.73)	
*Education *				
Uneducated	571 (87.31)		83 (12.69)	<0.001
Educated (≥5 years)	644 (96.55)		23 (3.45)	
*Monthly income (PKR)*				
Low income (≤8500)	280 (92.11)		24 (7.89)	0.925
Middle income (8501–103900)	935 (91.94)		82 (8.06)	
*Occupation/work status*				
Unemployed	752 (91.93)		66 (8.07)	0.990
Employed (skilled)	236 (92.19)		20 (7.81)	
Labor (unskilled)	227 (91.90)		20 (8.10)	
*Smoking status*				
Never smoked	938 (93.24)		68 (6.76)	0.009
Ex-smoker	85 (86.73)		13 (13.27)	
Current smoker	192 (88.48)		25 (11.52)	

Comorbidity is the presence of one or more of the following conditions: type 2 DM, hypertension, CVD, hypercholesterolemia, OA, and CA. The reported *p* values are for chi-square tests of independence for categorical data.

**Table 2:** Overall and gender-wise multivariate logistic regression analysis of the participants' characteristics (both BMI international and Asian cut-offs, WC and WHR categories) associated with having self-reported comorbidity.

	Overall		Multivariate analysis		Multivariate analysis	
	Multivariate analysis		Men		Women	
	Odds ratio	*p* value	Odds ratio	*p* value	Odds ratio	*p* value
	[95% CI]		[95% CI]		[95% CI]	
*BMI category (international cut-off)*						
Underweight	0.99 [0.42,2.32]	0.978	1.12 [0.37,3.45]	0.836	0.75 [0.19,2.98]	0.689
Normal weight	1	—	1	—	1	—
Overweight	1.89 [1.10,3.26]	0.021	1.14 [0.48,2.75]	0.762	2.64 [1.23,5.67]	0.013
Obesity	5.75 [2.84,11.64]	<0.001	8.49 [2.50,28.81]	0.001	5.19[2.03,13.25]	0.001
*BMI category (Asian cut-off)*						
Underweight	1.10 [0.45,2.66]	0.835	1.16 [0.36,3.66]	0.805	0.88 [0.21,3.67]	0.863
Normal weight	1	—	1	—	1	—
Overweight	1.38 [0.67,2.84]	0.382	1.19 [0.44,3.21]	0.728	1.52 [0.51,4.55]	0.453
Obesity	2.87 [1.66,4.97]	<0.001	1.96 [0.86,4.48]	0.109	3.73 [1.68,8.29]	0.001
*WC category*						
Normal weight	1	—	1	—	1	—
Overweight	1.20 [0.62,2.33]	0.588	0.95 [0.27,3.31]	0.933	1.20 [0.51,2.81]	0.669
Obesity	3.75 [2.06,6.85]	<0.001	6.38 [2.24,18.19]	0.001	3.37 [1.49,7.64]	0.004
*WHR category*						
Normal weight	1	—	1	—	1	—
Overweight	1.95 [0.84,4.51]	0.118	2.15 [0.81,5.70]	0.126	1.96 [0.06,68.90]	0.710
Obesity	1.72 [0.63,4.72]	0.292	3.93 [0.99,15.58]	0.052	1.06 [0.33,33.70]	0.975

CI: confidence interval, adjusted by gender, age, marital status, education, monthly income, occupation/work status, and smoking status.
